# Potential Sources of Racial and Ethnic Disparities in Nursing Home Influenza Vaccination

**DOI:** 10.1093/geroni/igab046.3198

**Published:** 2021-12-17

**Authors:** Melissa Riester, Elliott Bosco, Barbara Bardenheier, Patience Moyo, Rosa Baier, Melissa Eliot, Andrew Zullo

**Affiliations:** 1 Brown University, Providence, Rhode Island, United States; 2 Brown University School of Public Health, Smithfield, Rhode Island, United States; 3 Brown University School of Public Health, Providence, Rhode Island, United States

## Abstract

Racial and ethnic disparities in influenza vaccination among nursing home (NH) residents are well-documented and have persisted over time, suggesting that new strategies are necessary to reduce disparities. We conducted a retrospective cohort study to examine the degree to which observable characteristics drove influenza vaccination disparities. We linked Minimum Data Set (MDS) assessments to facility-level data for short- and long-stay NH residents aged ≥65 years. We included residents with six-month continuous enrollment in Medicare and an MDS assessment during the influenza season (October 1, 2013 through March 31, 2014). Using nonlinear Oaxaca-Blinder decomposition, we decomposed the disparities in vaccination between White versus Black and White versus Hispanic residents. We analyzed short- and long-stay residents separately. Our study included 630,373 short-stay and 1,029,593 long-stay residents. Among short-stay residents, 67.2% of Whites, 55.1% of Blacks, and 54.5% of Hispanics were vaccinated against influenza; among long-stay residents, 84.2% of Whites, 76.7% of Blacks, and 80.8% Hispanics were vaccinated against influenza. Across the four comparisons, the crude disparity in influenza vaccination ranged from 3.4-12.7 percentage points. By equalizing 27 characteristics, these disparities could be reduced by 37.7%-59.2%. Living in a predominantly White facility and proxies for NH quality were important contributors to the disparity, although characteristics unmeasured in our data (e.g., NH staff attitudes and beliefs) contributed 40.8%-62.3% to the disparity across comparisons. Intervening on factors associated with NH quality may reduce racial/ethnic disparities in influenza vaccination. Qualitative research is essential to explore potential contributors not captured in our administrative data.

